# Identification of transcobalamin deficiency with two novel mutations in the *TCN2* gene in a Chinese girl with abnormal immunity: a case report

**DOI:** 10.1186/s12887-020-02357-6

**Published:** 2020-10-06

**Authors:** Shihong Zhan, Fangfang Cheng, Hailong He, Shaoyan Hu, Xing Feng

**Affiliations:** 1 The Neonatal Department, Children’s Hospital of Soochow University, No. 92 Zhongnan Road, 215000 Suzhou, Jiangsu Province China; 2 Infectious Diseases Department, Children’s Hospital of Soochow University, No. 92 Zhongnan Road, 215000 Suzhou, Jiangsu Province China; 3 Hematology-Oncology Department, Children’s Hospital of Soochow University, No. 92 Zhongnan Road, 215000 Suzhou, Jiangsu Province China

**Keywords:** Cobalamin, deficiency, transcobalamin, megaloblastic anaemia, case report

## Abstract

**Background:**

Transcobalamin (TC) transports vitamin B12 from blood into cells. TC II deficiency is a rare autosomal recessive disorder. It is characterized by failure to thrive, diarrhoea, pallor, anaemia, pancytopenia or agammaglobulinemia. It is usually confirmed by molecular analysis of the *TCN2* gene. We report a 2-month-old girl with two novel mutations, which were first reported in humans.

**Case presentation:**

We present a 2-month-old Chinese girl with pancytopenia, severe combined immunodeficiency disease, and megaloblastic anaemia. Targeted next-generation sequencing (NGS) was performed, which detected compound heterozygous variants in exon 7 of the *TCN2* gene (Mutation 1: c.1033 C > T; Mutation 2: c.1017-1031delinsGTAACAGAGATGGTT). These mutations result in stop codons in TCN2. The c.1033C > T mutation causes a stop at codon 345 (p.Gln345Ter), and the c.1017-1031delinsGTAACAGAGATGGTT mutation causes a stop at codon 340 (p.Leu340Ter). After being diagnosed, she was treated with intramuscular 1 mg hydroxycobalamin (OH-Cbl) every day for 2 months. The CBC value returned to normal after half a month. The peripheral blood lymphocyte subsets and immunoglobulin recovered after 2 months. Then, the dosage of OH-Cbl was gradually reduced.

**Conclusions:**

TC II deficiency is a serious complication that requires lifelong treatment. Its diagnosis is difficult due to the lack of clearly identifiable symptoms. Genetic testing should be performed as early as possible if this disease is suspected. The specific observations of this case report make a considerable contribution to the literature and provide a reference for the diagnosis and treatment of future cases.

## Background

Transcobalamin (TC), a vitamin B12 (cobalamin, Cbl) binding protein in plasma, promotes the cellular uptake of vitamin B12 by receptor-mediated endocytosis. Inherited TC II deficiency is an autosomal recessive disorder characterized by megaloblastic anaemia caused by cellular vitamin B12 depletion [1, 2]. It may be accompanied by neurological complications, including a delay in psychomotor and mental development. Sometimes severe immune deficiency, including abnormal humoral and cellular immunity, is also observed.

The protein (TC II) is encoded by the *TCN2* gene, which spans 18 kb and contains 9 exons on chromosome 22q12. b. TC II deficiency was first reported in two siblings in 1971, and since then, fewer than 50 affected individuals have been identified (Table 1) [2–6]. The reported mutations in the *TCN2* gene include deletions or insertions, nonsense mutations, and point mutations. Of these, deletions or insertions are the most common, causing frameshifts that result in protein truncation [4, 5, 7, 8]. A few polymorphic variants have also been reported [8].

**Table 1:** Summary of the mutations reported in the *TCN2* gene

Exon	gDNA	Effect	Reference
2	c.67A > G	p.Ile23 Val	Afman et al 2002 [9]
8	c.1196G > A	p.Arg399Gln	Afman et al 2002
5	c.703dupA	p.Thr235Asn fs*69	Bartakke et al 2015 [10]
8	c.1127T > C	p.Leu376Ser	Grarup et al 2013 [11]
4	c.580 + 624A > T	p.?	Häberle et al 2009 [12]
7	c.940 + 303_c.1106 + 746delinsCTGG	p.?	Häberle et al 2009
8	c.1194C > T	p.Arg399Ter	Khera et al 2019 [13]
2	c.172delC	p.Leu58Tyrfs*28	Li et al 1994 [4]
3	c.387delA	p.Gln130Serfs*77	Li et al 1994
6	c.927-930del	p.Cys309Trp fs*50	Li et al 1994
8	c.1110T > G	p.Tyr370Term	Li et al 1994
1–9	large deletion involving all but 3' end of gene	p.?	Li et al 1994
3	c.427 + 2 T > G	p.?	Namour et al 2003 [7]
1	c.64 + 4A > T	p.?	Nashabat et al 2017 [14]
7	c.1106 + 1G > A	p.Met315fs	Nissen et al 2010 [15]
8	deletion ex. 8	p.?	Nissen et al 2010
8	c.1195C > T	p.Arg399Term	Prasad et al 2008
5	c.679C > T	p.Arg227Term	Pupavac et al 2016 [16]
1	c.62G > A	p.Cys21Tyr	Qian et al 2002 [17]
1	c.31C > G	p.Leu11Val	Qian et al 2002
2	c.145C > T	p.His49Tyr	Qian et al 2002
2	c.254T > A	p.Leu85Gln	Qian et al 2002
2	c.79G > C	p.Asp27Asn	Qian et al 2002
2	c.257G > A	p.Gly86Glu	Qian et al 2002
3	c.330dupC	p.Ala111Argfs*7	Ratschmann et al 2009 [6]
4	c.580 + 1G > C	p.?	Schiff et al 2010 [18]
4	c.501_503del	p.Leu167del	Schiff et al 2010
8	c.1139dupA	p.Tyr380Ter	Schiff et al 2010
8	c.1117_1118del	p.Gln373Glyfs*38	Schiff et al 2010
9	c.1236_1237del	p.Tyr412fs	Schiff et al 2010
3	c.423del	p.Ile142Leufs*65	Trakadis et al 2014 [19]
3	c.348_349del	p.Cys116fs	Trakadis et al 2014
4	c.472G > T	p.Gly158Cys	Trakadis et al 2014
4	c.497_498del	p.Leu166Profs*7	Trakadis et al 2014
5	c.745del	p.Ala249Hisfs*6	Trakadis et al 2014
6	c.937C > T	p.Arg313Ter	Trakadis et al 2014
7	c.940 + 283_940 + 286del	p.?	Trakadis et al 2014
7	c.940 + 303_1106 + 764delinsCTGG	p.?	Trakadis et al 2014
7	c.1013_1014 delinsTAA	p.Ser338Ilefs*27	Trakadis et al 2014
8	c.1106 + 1516_1222 + 1231del	p.?	Trakadis et al 2014
1	c.106C > T	p.Gln36Term	Ünal et al 2015 [20]
8	c.1107 − 347_1222 + 981delins 364 bp	p.?	Ünal et al 2015
7	c.1017-1031delinsGTAACAGAGATGGTT	p.Leu340Ter	Novel this paper
7	c. 1033C > T	p.Gln345Ter	Novel this paper

Overall, patients with TC II deficiency can present with variable clinical features, including failure to thrive (FTT), diarrhoea, pallor, and anaemia. Many mutations in the *TCN2* gene have been reported that are related to the disease. Here, we present a 2-month-old Chinese girl with pancytopenia, severe combined immunodeficiency disease, and megaloblastic anaemia with novel compound heterozygous variants in the *TCN2* gene to emphasize the importance of early diagnosis and treatment.

## Case presentation

A 2-month-old girl presented with cough for three days and was diagnosed with pancytopenia based on her initial test results. Her mother had gestational hypertension and was treated with oral labetalol. No abnormalities were found on prenatal ultrasound, and the foetal heart rate was normal. She was born naturally at 38 weeks with a 9-9-10 Apgar score. Amniotic fluid is clear. Her birth weight was 3000 g, her body length was 48 cm, and her head circumference was 33 cm. All of her results were between 50% and 75% of the same gestational age 21. There was no family history of similar diseases, especially of the haematologic system. Both parents and her 10-year-old sister were in good health.

**Fig. 1:**
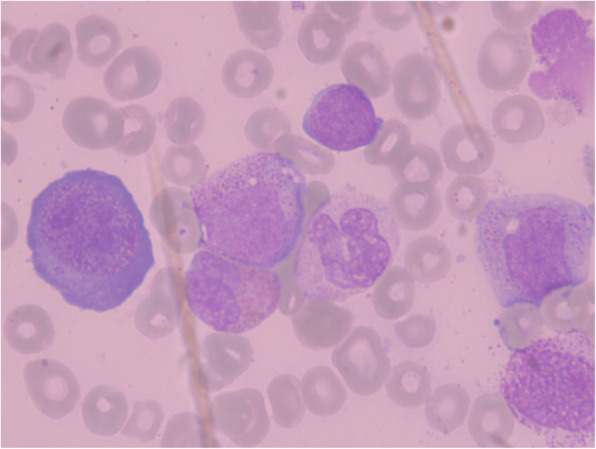
Bone marrow aspiration: megaloblastic changes and dysplasia in erythrocytic and megakaryocytic lineages (Pappenheim staining: magnification 1000)

Weight gain during the neonatal period was within the normal range. On the 38th day of physical examination, her weight was 4 kg. Her complete blood count (CBC) haemoglobin (Hb) level was 87 g/L (reference: 110–140 g/L), her red blood cell (RBC) count was 2.67 × 10^12^/L (reference: 3.5-5.0 × 10^12^/L), and her white blood cell (WBC) and platelet (Plt) counts were normal. She was believed to have physiological anaemia and was not treated. She began coughing and vomiting on day 52. A second CBC revealed pancytopenia with a WBC count of 4.5 × 10^9^/L (reference: 4.0–10.0 × 10^9^/L), Hb level of 58 g/L, Plt count of 46 × 10^9^/L (reference: 100.0-300.0 × 10^9^/L), mean corpuscular volume (MCV) of 106 fL (reference: 80–100 fL), mean corpuscular Hb (MCH) of 33.5 pg/cell (reference: 27–34 pg/cell), and red blood cell volume distribution width (RDW) of 24.6% (reference: 11.6%-16.5%). Peripheral blood lymphocyte subsets indicated high CD3 + cells 93.8% (reference: 39.0%-73.0%) and low CD3-CD(16 + 56) + cells 0.4% (reference: 3.0%-16.0%) and low CD3-CD19 + cells 4.9% (reference: 7.0%-41.0%), which indicated the abnormal differentiation of lymphocytes. Similarly, a humoral immunoassay showed extremely low levels of globulin in the blood, with 0.05 g/L immunoglobulin IgA (reference: 0.13–0.35 g/L), 0.46 g/L IgG (reference: 3.22–7.18 g/L), and 0.04 g/L IgM (reference: 0.23–0.91 g/L). Cobalamin and folic acid levels in peripheral blood were in the normal range. Bone marrow examination revealed megaloblastic changes and dysplasia in erythrocytic and megakaryocytic lineages with no blasts (Fig. 1).

The infant was admitted to our hospital. She received supportive treatment including blood transfusions and G-CSF injections, parenteral nutrition, and antibiotic treatment with meropenem and vancomycin for her pneumonia. However, her pulmonary infection was difficult to control. Diarrhoea and weight stagnation also appeared during hospitalization. Respiratory failure occurred on day 98, and she required tracheal intubation with ventilator-assisted ventilation.

**Fig. 2:**
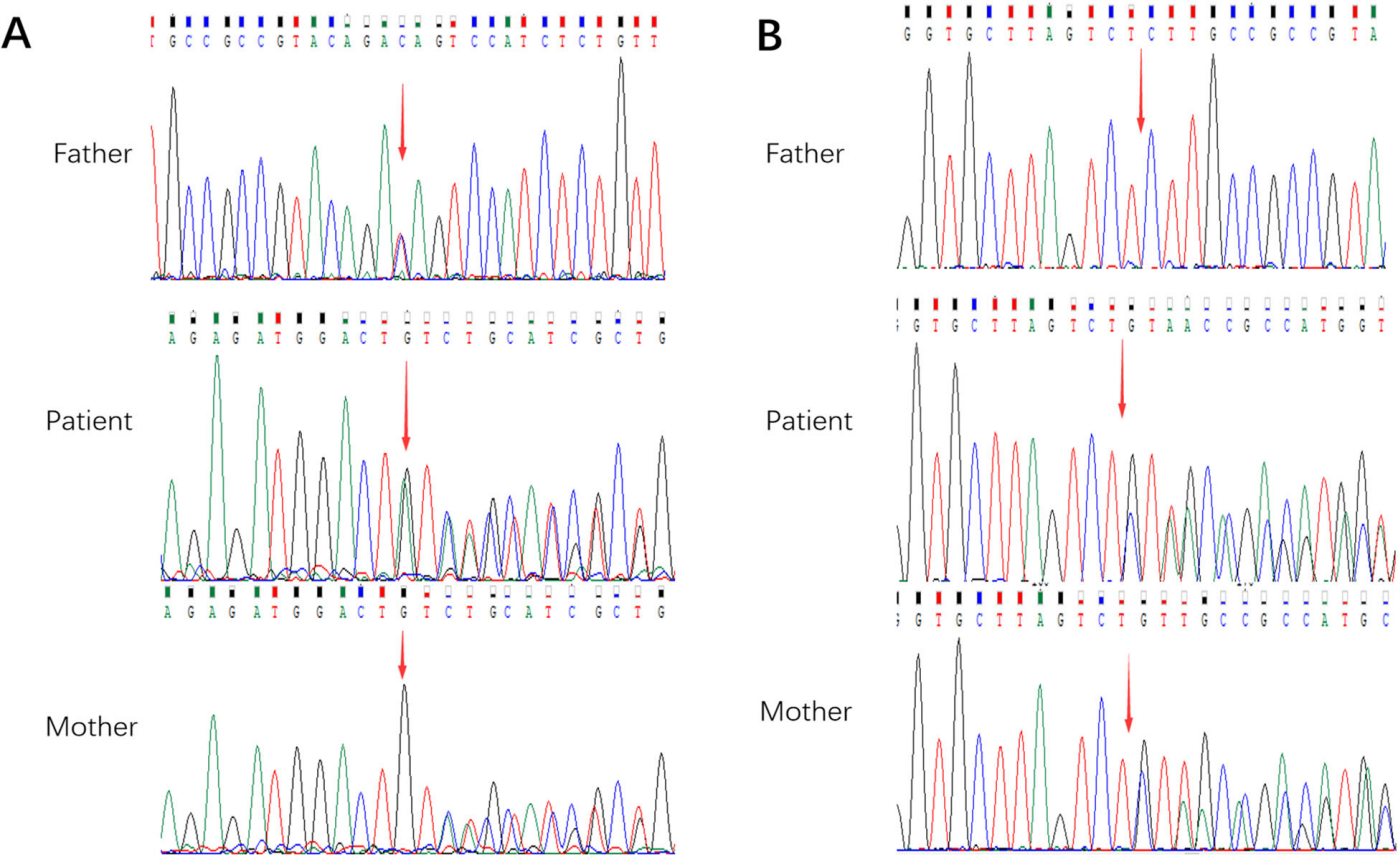
Sequencing results of the *TCN2* mutation. **a**. Mutation 1: c.1033 C > T; **b**.Mutation 2: c.1017-1031delinsGTAACAGAGATGGTT.

Targeted next-generation sequencing was performed on the patient. The gene panel (Mygenostics) contained 816 genes related to hereditary blood disease, which identified compound heterozygous variants in exon 7 of the *TCN2* gene. The targeted resequencing data were confirmed by Sanger sequencing. Parental genetic tests were used to determine whether this mutation was inherited or new. A paternally inherited rare variant (Mutation 1: hg19, chr22-31013409, NM_000355, c. 1033C > T, p.Gln345Ter, not present in 1000 Genomes, not present in esp6500, not present in gnomAD, 0.00001647 in ExAC_ALL, 0.0001 in ExAC_EAS) and a maternally inherited variant (Mutation 2: hg19, chr22:31013392–31013407, NM_000355, c.1017-1031delinsGTAACAGAGATGGTT, p.Leu340Ter, not present in 1000 Genomes, esp6500, gnomAD and ExAC) were identified in the patient. These mutations result in stop codons of *TCN2*. The c.1033C > T causes a stop at codon 345 (p.Gln345Ter), and the c.1017-1031delinsGTAACAGAGATGGTT causes a stop at codon 340 (p.Leu340Ter) (Fig. 2).

After being diagnosed with TC II deficiency, she was treated with intramuscular 1 mg OH-Cbl every day for 2 months, and subsequently, the dosage was gradually reduced to once every 4 weeks. The CBC value returned to normal after half a month of OH-Cbl administration: WBC, 6.02 × 10^9^/L; Hb level, 128 g/L; Plt, 185 × 10^9^/L; MCV, 82 fL; MCH, 27.5 pg/cell; and RDW, 13.9%. Her pneumonia was obviously improved, and she was weaned from the ventilator. She then had a brain MRI, which showed mild encephalatrophy. After 2 months of OH-Cbl administration, the peripheral blood lymphocyte subsets and immunoglobulin also recovered. No obvious side effects were observed during the treatment. The girl is now 22 months old, 85 cm in height, and 12 kg in weight. She walked at 14 months and began speaking at 18 months.

## Discussion

**Fig. 3:**
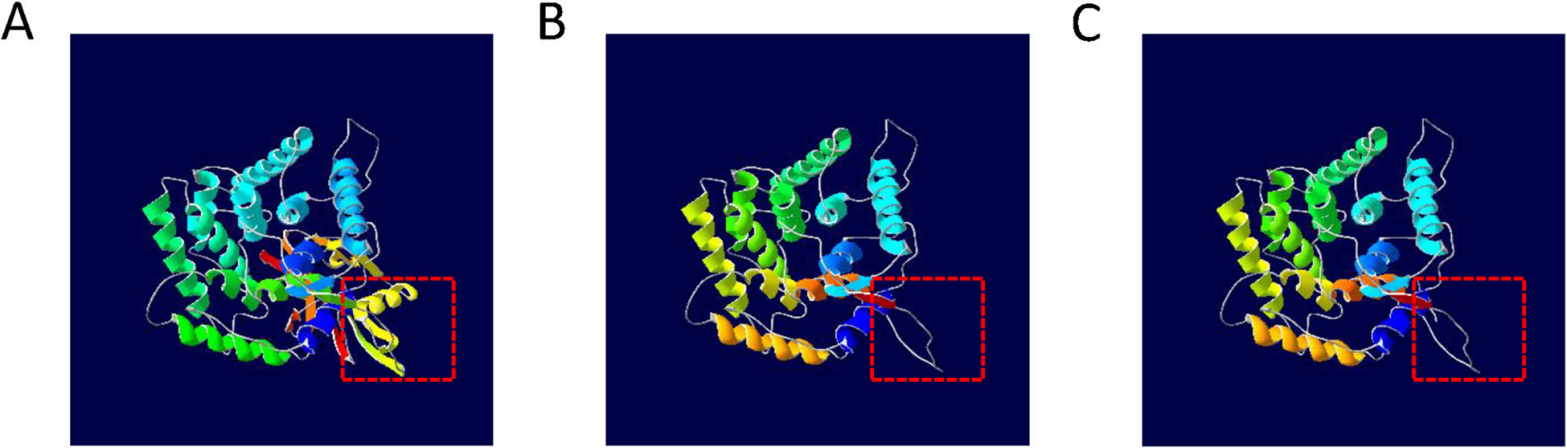
Protein structure prediction caused by compound heterozygous gene mutations of the TCN2 gene. **a**, The three-dimensional structure diagram of the wild-type TCN2 protein; the dashed box shows the C-terminal domain. **b**, The mutant of TCN2 p.Leu340Ter, the dashed box shows the lost C-terminal domain. **c**, The mutant of TCN2 p.Gln345Ter; the dashed box shows the lost C-terminal domain.

Most pathogenic mutations are predicted to result in extremely low levels or complete absence of TC II. TC II deficiency eventually leads to the gradual depletion of intracellular cobalamin storage in the first few weeks after birth and causes secondary damage of methionine synthase and methyl-malonyl COA mutase activities [22]. There is considerable genetic heterogeneity of TC II deficiency, as evidenced by the presence of nonsense mutations, deletions, RNA editing, and a mutation affecting a splice site (details of mutations are provided in Table 1). However, previous studies have not reported any genotype–phenotype correlations. Here, we report 2 new mutations (p.Gln345Ter and p.Leu340Ter) in the *TCN2* gene that result in TC II deficiency. The wild-type *TCN2* transcript NM000355 encodes a member of the vitamin B12-binding protein family with 427 amino acids; however, both of these mutations lead to early termination of the codon, which may cause nonsense-mediated mRNA decay (NMD) or result in transamin II protein with truncated amino acid sequences. Due to NMD or truncations, mutated TCN2 may lose its function, leading to transcobalamin deficiency in the patient. These two mutations were not reported in the HGMD database, which indicated that they are novel mutations, and this is the first report in humans; this finding has expanded the mutation spectrum of TCN2. We obtained *TCN2* gene sequences (NM_000355) from NCBI Gene and used SWISS-MODEL, an automated protein homology modelling server, to generate three-dimensional structures of *TCN2* wild-type and mutant (p.L340X and p.Q345X) protein [23–25]. Both mutants showed loss of an unknown C-terminal domain (with unknown function) of transcobalamin, a vitamin B12-binding protein that transports cobalamin into cells (Fig. 3) [26].

We report the case of a two-month-old baby girl with vomiting, diarrhoea, weight stagnation, pancytopenia and combined immunodeficiency. She received intensive care in the Paediatric Intensive Care Unit for three months. Tarkadis et al. summarized the clinical characteristics of 24 patients diagnosed with TC II deficiency [19]; the clinical features included individuals with blood complications such as anaemia or pancytopenia (87.5%, 21/24); glossitis (16.7%, 4/24); skin manifestations such as nonspecific rash, purpura, and petechiae (12.5%, 3/24); individuals with failure to thrive (66.7%, 16/24); gastrointestinal complications such as vomiting and diarrhoea (37.5%, 9/24); neurological symptoms such as weakness, hypotonia, myoclonic-like movements, or delayed milestones (29.2%, 7/24); immunological abnormalities such as agammaglobulinemia, low IgG, or low T and B cell counts (16.7%, 4/24), and recurrent infections (8.3%, 2/24) [19]. Almost all these symptoms appear between 2 and 4 weeks after birth and worsen over approximately 2 months. Early diagnosis is very difficult because there are no typical clinical manifestations of TC II deficiency, and multiple systems can be involved. The blood system is most often affected and could be an indication. However, several other diseases also lead to pancytopenia in a newborn or small baby. These include disorders that cause increased blood cell damage, such as systemic autoimmune disorders, immune dysregulation syndrome, systemic lupus erythematosus, disseminated intravascular coagulation, and hypersplenism, and those resulting in haematopoietic function failure include a variety of infections, infiltrative bone marrow diseases, and other genetic factors. Among these, the most common is infection caused by Epstein-Barr virus, cytomegalovirus, chicken pox, rickettsia, and bacterial sepsis. TC II deficiency is extremely rare and prone to misdiagnosis. The diagnosis delay causes progressive deterioration in the patient and increases the chance of infection and the need for blood transfusion. However, if the patient is diagnosed in the neonatal period and receives early treatment, there are chances of better prognosis, especially with respect to the development of the nervous system and growth. Therefore, early recognition and treatment of the disease are particularly important.

With the development of diagnostic technology, at present, the diagnosis of this disease clearly depends on next-generation sequencing. TC II deficiency has also been reportedly identified by tandem mass spectrometry. The flagged values were for C3 and C3/C2 acyl carnitine elevations [27]. However, these reports are not entirely consistent [19]. The transcobalamin-vitamin B12 complex, known as Holo-TC, is metabolically active cobalamin; in the case of TC II deficiency, Holo-TC is undetectable [28]. The treatment of TC II deficiency mainly consists of hydroxycobalamin (OH-Cbl) or cyanocobalamin (CN-Cbl) administration. Although there was no significant difference in efficacy between the two treatments, the mode of administration seemed to affect the prognosis, and intramuscular injection was recommended [19]. The patient requires treatment for life, and the most common complications include speech and attention disorders. The patient we described herein was prescribed 1 mg OH-Cbl intramuscularly every day on day 122, and her CBC value returned to normal after half a month. After a treatment period of 2 months, OH-Cbl gradually changed to once every 4 weeks. As a result of earlier targeted treatment, she achieved good therapeutic effects.

In summary, TC II deficiency is a rare autosomal recessive disorder that requires lifelong treatment. Early recognition and treatment of the disease is particularly important. It should be ruled out for infants diagnosed with pancytopenia and/or showing developmental delay. This study reports two novel mutations in the *TCN2* gene that result in mutated proteins with possible loss of function. We believe that the specific observations made during this case report will provide a reference for the diagnosis and treatment of future cases.

## Data Availability

The datasets used during the current study are available from the corresponding author upon reasonable request.

## Abbreviations

TC: Transcobalamin
NGS: Next-generation sequencing
Cbl: Cobalamin
OH-Cbl: Hydroxycobalamin
CN-Cbl: Cyanocobalamin

## References

[1] Hakami N, Neiman PE, Canellos GP (1971). Neonatal megaloblastic anemia due to inherited transcobalamin II deficiency in two siblings. N Engl J Med.

[2] Regec A, Quadros EV, Platica O (1995). The cloning and characterization of the human transcobalamin II gene. Blood.

[3] Arwert F, Porck HJ, Frater-Schröder M (1986). Assignment of human transcobalamin II (TC2) to chromosome 22 using somatic cell hybrids and monosomic meningioma cells. Hum Genet.

[4] Li N, Rosenblatt DS, Kamen BA (1994). Identification of two mutant alleles of transcobalamin II in an affected family. Hum Mol Genet.

[5] Li N, Rosenblatt DS, Seetharam B (1994). Nonsense mutations in human transcobalamin II deficiency. Biochem Biophys Res Commun.

[6] Ratschmann R, Minkov M, Kis A (2009). Transcobalamin II deficiency at birth. Mol Genet Metab.

[7] Namour F, Helfer AC, Quadros EV (2003). Transcobalamin deficiency due to activation of an intra exonic cryptic splice site. Br J Haematol.

[8] Watkins D, Rosenblatt DS (2011). Inborn errors of cobalamin absorption and metabolism. Am J Med Genet C Semin Med Genet.

[9] Afman LA, Lievers KJ, van der Put NM (2002). Single nucleotide polymorphisms in the transcobalamin gene: relationship with transcobalamin concentrations and risk for neural tube defects. Eur J Hum Genet.

[10] Bartakke S, Saindane A, Udgirkar V (2015). Novel Mutation in an Indian Patient with Transcobalamin II Deficiency. Indian J Pediatr.

[11] Grarup N, Sulem P, Sandholt CH (2013). Genetic architecture of vitamin B12 and folate levels uncovered applying deeply sequenced large datasets. PLoS Genet.

[12] Häberle J, Pauli S, Berning C (2009). TC II deficiency: avoidance of false-negative molecular genetics by RNA-based investigations. J Hum Genet.

[13] Khera S, Pramanik SK, Patnaik SK (2019). Transcobalamin deficiency: vitamin B12 deficiency with normal serum B12 levels. BMJ Case Rep.

[14] Nashabat M, Maegawa G, Nissen PH (2017). Long-term Outcome of 4 Patients With Transcobalamin Deficiency Caused by 2 Novel TCN2 Mutations. J Pediatr Hematol Oncol.

[15] Nissen PH, Nordwall M, Hoffmann-Lücke E (2010). Transcobalamin deficiency caused by compound heterozygosity for two novel mutations in the TCN2 gene: a study of two affected siblings, their brother, and their parents. J Inherit Metab Dis.

[16] Pupavac M, Tian X, Chu J, et al. Added value of next generation gene panel analysis for patients with elevated methylmalonic acid and no clinical diagnosis following functional studies of vitamin B12 metabolism. Mol Genet Metab. 2016;117(3):363–8. doi: 10.1016/j.ymgme.2016.01.008 [published Online First: 2016/02/02]. 10.1016/j.ymgme.2016.01.008 26827111

[17] Qian L, Quadros EV, Regec A, et al. Congenital transcobalamin II deficiency due to errors in RNA editing. Blood Cells Mol Dis. 2002;28(2):134–42. doi:https://doi.org/10.1006/bcmd.2002.0499 [published Online First: 2002/06/18]. discussion 43 – 5. 10.1006/bcmd.2002.0499 12064907

[18] Schiff M, De Baulny HO, Bard G (2010). Should transcobalamin deficiency be treated aggressively?. J Inherit Metab Dis.

[19] Trakadis YJ, Alfares A, Bodamer OA (2014). Update on transcobalamin deficiency: clinical presentation, treatment and outcome. J Inherit Metab Dis.

[20] Ünal Ş, Rupar T, Yetgin S (2015). Transcobalamin II deficiency in four cases with novel mutations. Turk J Haematol.

[21] enton TR, Kim JH (2013). A systematic review and meta-analysis to revise the Fenton growth chart for preterm infants. BMC Pediatr.

[22] Gherasim C, Lofgren M, Banerjee R (2013). Navigating the B(12) road: assimilation, delivery, and disorders of cobalamin. J Biol Chem.

[23] Biasini M, Bienert S, Waterhouse A, et al. SWISS-MODEL: modelling protein tertiary and quaternary structure using evolutionary information. *Nucleic Acids Res* 2014;42(Web Server issue):W252–8. doi: 10.1093/nar/gku340 [published Online First: 2014/05/02]. 10.1093/nar/gku340 PMC4086089 24782522

[24] Guex N, Peitsch MC, Schwede T (2009). Automated comparative protein structure modeling with SWISS-MODEL and Swiss-PdbViewer: a historical perspective. Electrophoresis.

[25] Kiefer F, Arnold K, Künzli M (2009). The SWISS-MODEL Repository and associated resources. Nucleic Acids Res.

[26] Johnston J, Bollekens J, Allen RH (1989). Structure of the cDNA encoding transcobalamin I, a neutrophil granule protein. J Biol Chem.

[27] Prasad C, Cairney AE, Rosenblatt DS (2012). Transcobalamin (TC) deficiency and newborn screening. J Inherit Metab Dis.

[28] Wolffenbuttel BH, Wouters HJ, Heiner-Fokkema MR, et al. The many faces of cobalamin (vitamin B12) deficiency. *Mayo Clinic Proceedings: Innovations, Quality & Outcomes* 2019;3(2):200–14. doi: 10.1016/j.mayocpiqo.2019.03.002. 10.1016/j.mayocpiqo.2019.03.002 PMC6543499 31193945

